# IL-6 in Osteoarthritis: Effects of Pine Stilbenoids

**DOI:** 10.3390/molecules24010109

**Published:** 2018-12-29

**Authors:** Mirka Laavola, Tiina Leppänen, Mari Hämäläinen, Katriina Vuolteenaho, Teemu Moilanen, Riina Nieminen, Eeva Moilanen

**Affiliations:** 1 The Immunopharmacology Research Group, Faculty of Medicine and Health Technology, Tampere University and Tampere University Hospital, 33014 Tampere, Finland; laavola.mirka.m@student.uta.fi (M.L.); tiina.m.leppanen@uta.fi (T.L.); mari.j.hamalainen@uta.fi (M.H.); katriina.vuolteenaho@uta.fi (K.V.); teemu.moilanen@coxa.fi (T.M.); riina.m.nieminen@uta.fi (R.N.); 2 Coxa Hospital for Joint Replacement, 33101 Tampere, Finland

**Keywords:** interleukin-6, osteoarthritis, stilbenoids, pinosylvin, aggrecan, chondrocytes

## Abstract

Interleukin-6 (IL-6) is involved in the pathogenesis of various inflammatory diseases, like rheumatoid arthritis (RA). In the present study, we investigated the role of IL-6 in osteoarthritis (OA) patients and the effects of the stilbenoids monomethyl pinosylvin and pinosylvin on the expression of the cartilage matrix components aggrecan and collagen II and the inflammatory cytokine IL-6 in human OA chondrocytes. Synovial fluid and plasma samples were obtained from 100 patients with severe OA [BMI 29.7 (8.3) kg/m^2^, age 72 (14) years, median (IQR); 62/38 females/males] undergoing total knee replacement surgery. IL-6 and matrix metalloproteinase (MMP) concentrations in synovial fluid and plasma were measured by immunoassay. The effects of pinosylvin on the expression of IL-6, aggrecan, and collagen II were studied in primary cultures of human OA chondrocytes. IL-6 levels in synovial fluid from OA patients [119.8 (193.5) pg/mL, median (IQR)] were significantly increased as compared to the plasma levels [3.1 (2.7) pg/mL, median (IQR)] and IL-6 levels in synovial fluid were associated with MMPs and radiographic disease severity. Natural stilbenoids monomethyl pinosylvin and pinosylvin increased aggrecan expression and suppressed IL-6 production in OA chondrocytes. The results propose that IL-6 is produced within OA joints and has an important role in the pathogenesis of OA. Stilbenoid compounds monomethyl pinosylvin and pinosylvin appeared to have disease-modifying potential in OA chondrocytes.

## 1. Introduction

Interleukin-6 (IL-6) was cloned in 1980s and it was first shown to promote the activation of T and B lymphocytes as well as to regulate the inflammation-associated acute-phase response. Currently, IL-6 is known as a mediator of inflammation, immune response and hematopoiesis [1]. Targeting IL-6 has become important in the drug development because of the pathological role of IL-6 in numerous adverse conditions. Tocilizumab which is a humanized monoclonal antibody against IL-6 receptor, is used as a second-line treatment of rheumatoid arthritis (RA). 

Osteoarthritis (OA) is the most common form of arthritis. The etiology of OA is still largely unknown although risk factors like certain genes, gender, age, joint trauma and obesity have been identified. Nowadays, there are no effective disease-modifying treatments except surgical interventions and the treatment is mainly limited to analgesics and other symptomatic approaches [2,3]. 

**Figure 1:**
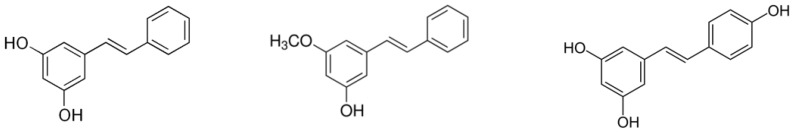
Chemical structures of pinosylvin, monomethyl pinosylvin and resveratrol.

IL-6 is detected in synovial fluid and expressed in osteoarthritic cartilage which makes its inhibition an appealing potential target in the treatment of OA [4,5,6,7]. Recently it was published that inhibition of IL-6 by tocilizumab reduced pain behavior in a monosodium iodoacetate-induced experimental model of OA in the rat, however, no clinical studies with IL-6 inhibitors in OA have been conducted to date [8]. Therefore, we were interested in studying the effects of the natural stilbenoids monomethyl pinosylvin and pinosylvin (Figure 1) in OA chondrocytes. Our hypothesis was supported by the fact that stilbenoids had been previously shown to inhibit the production of pro-inflammatory cytokines, including IL-6, in activated macrophages. Furthermore, resveratrol, the best known stilbenoid, structurally close to pinosylvin, has been shown to inhibit IL-6 in primary human chondrocytes [9,10,11]. 

In the present study, we report IL-6 concentrations in synovial fluid and plasma samples from 100 OA patients undergoing total knee replacement surgery and the association between the levels of IL-6, matrix metalloproteinases (MMPs) and the radiographic severity of the disease. In addition, we investigated the effects of monomethyl pinosylvin and pinosylvin on the expression of IL-6, aggrecan and collagen II in primary human OA chondrocytes. 

## 2. Results

### 2.1. IL-6 Concentrations in Synovial Fluid are Higher than those in Plasma in Patients with OA

In osteoarthritis patients (*n* = 100) undergoing knee replacement surgery, IL-6 concentrations in synovial fluid [119.8 (193.5) pg/mL, median (IQR)] were significantly higher than those in plasma [3.1 (2.7) pg/mL, median (IQR)]. No correlation between the synovial fluid and plasma levels were found suggesting that IL-6 is produced locally within the joint. 

### 2.2. IL-6 Concentrations in Synovial Fluid Correlate with the Radiographic Severity of OA and with Matrix Metalloproteinase Concentrations

**Figure 2:**
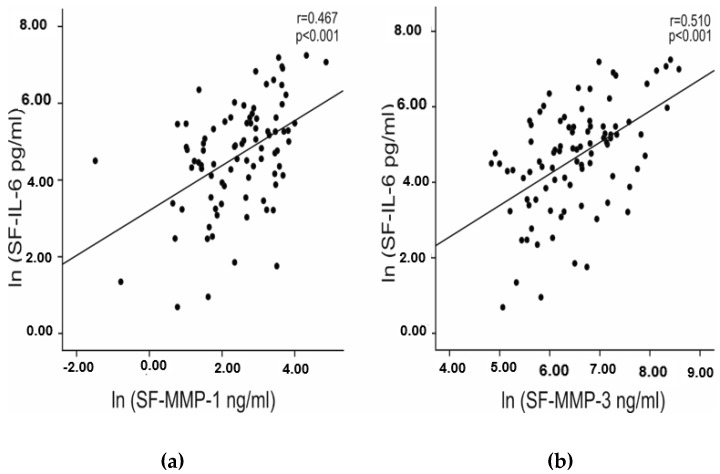
Correlation between IL-6 and MMP-1 (**a**) and MMP-3 (**b**) in patients with osteoarthritis. IL-6 and MMP levels in synovial fluid (SF) were measured by immunoassay. Natural logarithms (LN) of the SF concentrations of IL-6 and MMPs were calculated in order to have normally distributed variables for the Pearson correlation analysis. In the Figure, correlation coefficients (r) and p values are given. Synovial fluid samples were collected from 100 patients with knee OA [BMI 29.7 (8.3) kg/m^2^, age 72 (14) years, median (IQR); 62/38 females/males].

The preoperative knee radiographs were evaluated and Ahlbäck classification from grades 1 to 5 was used. Grades 1–3 and 4–5 were combined for the analysis. Mean synovial fluid IL-6 concentrations were higher (*p* = 0.027) in the group of grades 4 and 5 [234.1 (264.7) pg/mL, median (IQR)] than in the group of grade 1–3 [94.6 (183.0) pg/mL, median (IQR)] suggesting that IL-6 concentrations in synovial fluid are related to the disease severity. Furthermore, IL-6 in synovial fluid correlated with cartilage degrading matrix metalloproteinases MMP-1 (r = 0.467, *p* < 0.001) and MMP-3 (r = 0.510, *p* < 0.001) (Figure 2). 

### 2.3. Monomethyl Pinosylvin, Pinosylvin and Resveratrol Suppress IL-6 Expression in Primary Cultures of OA Chondrocytes

**Figure 3:**
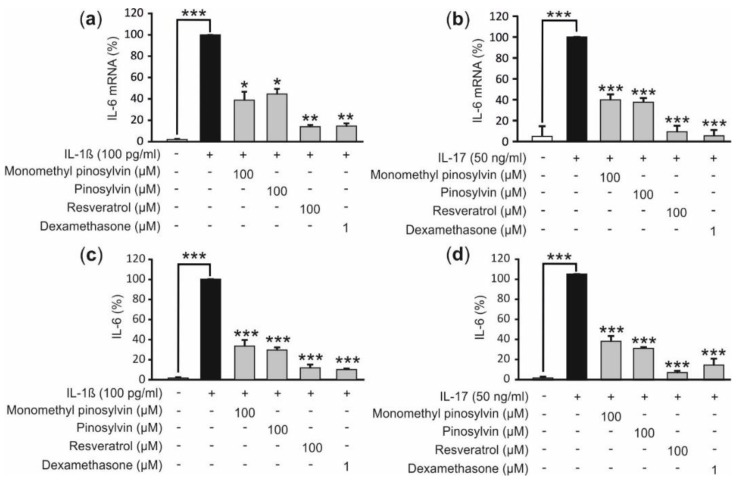
Effects of monomethyl pinosylvin, pinosylvin, resveratrol and the anti-inflammatory control compound dexamethasone in IL-1β and IL-17 stimulated primary human OA chondrocytes on IL-6 expression at mRNA (**a**,**b**) and protein (**c**,**d**) level at time point 24 h. IL-6 mRNA was measured by quantitative reversed transcriptase polymerase chain reaction (RT-PCR) and the results were normalized against GAPDH mRNA. IL-6 concentrations in the culture media were measured by immunoassay. IL-6 levels were 12.5–25.8 ng/mL in IL-1β and 2.6–10.2 ng/mL in IL-17 stimulated cells in the absence of the tested compounds. Primary chondrocytes were isolated from cartilage samples obtained from three consequent donors and the experiments were performed in duplicate; *n* = 3 was used in the calculations. Results are expressed as mean +SEM. * *p* < 0.05, ** *p* < 0.01 and *** *p* < 0.001 as compared to cells treated with IL-1β or IL-17 only.

Primary chondrocytes from OA patients produced IL-6 and that was significantly increased when the cells were exposed to the pro-inflammatory cytokine IL-1β or IL-17, both involved in the pathogenesis of OA [12]. Next, the effect of pine stilbenoids monomethyl pinosylvin and pinosylvin which are structurally close to the better known stilbenoid compound resveratrol were studied in cultures of primary human OA chondrocytes. Monomethyl pinosylvin and pinosylvin inhibited IL-6 expression at mRNA (Figure 3a,b) and protein level (Figure 3c,d) in both IL-1β and IL-17 stimulated chondrocytes as did the control stilbenoid resveratrol. Dexamethasone as a standard anti-inflammatory compound had an anticipated inhibitory effect also.

### 2.4. Pine Stilbenoids Inhibit NF-κB Mediated Transcription in Human Chondrocytes

**Figure 4:**
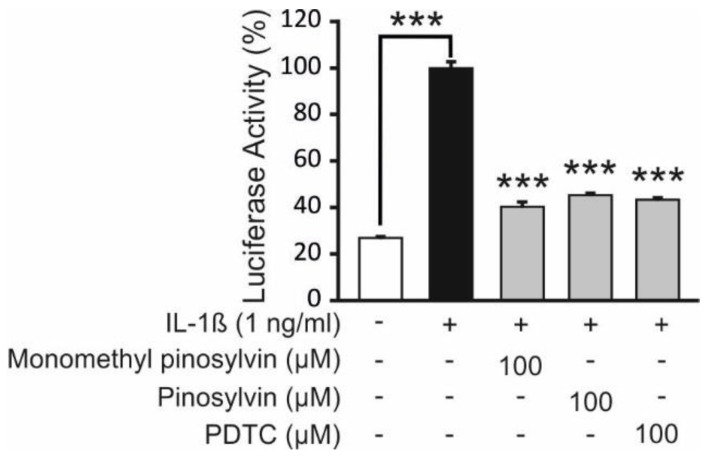
Effects of monomethyl pinosylvin and pinosylvin on NF-*k*B-mediated transcription in human T/C28a2 chondrocytes transfected with luciferase reporter construct. T/C28a2pGL4.32NFκB cells were stimulated with IL-1β in the presence of the pine stilbenoids or the known NF-κB inhibitor pyrrolidine dithiocarbamate (PDTC) for 5 h and luciferase activity was measured. Results are presented as mean +SEM, *n* = 4, *** *p* < 0.001 as compared to cells incubated with IL-1β only. The inhibitory effect was similar with ammonium pyrrolidine dithiocarbamate (PDTC), a known NF-κB inhibitor.

NF-κB is a key transcription factor regulating IL-6 production [1]. Therefore we investigated the effects of monomethyl pinosylvin and pinosylvin on NF-κB mediated transcription. T/C28a2 human chondrocyte cell line was engineered to express luciferase (LUC) gene under the control of an NF-κB driven promoter. Monomethyl pinosylvin and pinosylvin significantly inhibited NF-κB mediated transcription (measured as luciferase activity) (Figure 4). The inhibitory effect was similar with ammonium pyrrolidine dithiocarbamate (PDTC), a known NF-κB inhibitor. 

### 2.5. Pinosylvin, Monomethyl Pinosylvin and Resveratrol Enhance the Expression of the Anabolic Factor Aggrecan in Human Primary Chondrocytes

**Figure 5:**
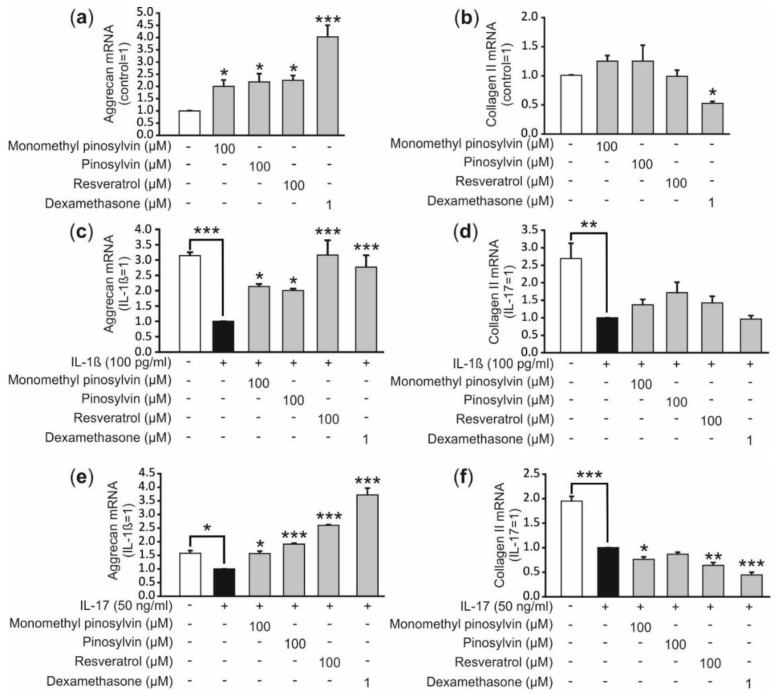
Effects of monomethyl pinosylvin, pinosylvin, resveratrol and the anti-inflammatory control compound dexamethasone on aggrecan and collagen II expression. Human primary chondrocytes were cultured with the tested compounds alone (**a**,**b**) or with IL-1β (**c**,**d**) or IL-17 (**e**,**f**) for 24 h before RNA was extracted. Aggrecan and collagen II mRNA was determined by reversed transcriptase polymerase chain reaction (RT-PCR) and the results were normalized against GAPDH mRNA. Primary chondrocytes were isolated from cartilage samples obtained from three consequent donors and the experiments were performed in duplicate; *n* = 3 was used in the calculations. Results are expressed as mean +SEM. * *p* < 0.05, ** *p* < 0.01 and *** *p* < 0.001 as compared to cells non-stimulated or treated with IL-1β or IL-17 only.

Aggrecan and collagen II are both major components of extracellular matrix in the cartilage [13]. IL-1β and IL-17 decreased the synthesis of the two anabolic factors in OA chondrocytes (Figure 5), as expected [12]. Interestingly, monomethyl pinosylvin, pinosylvin and resveratrol increased the aggrecan expression in non-stimulated cells and reversed the suppressive effect of IL-1β and IL-17 on aggrecan expression in OA chondrocytes but had no effect on collagen II expression (Figure 5). The control compound dexamethasone also enhanced aggrecan but not collagen expression.

## 3. Discussion

In OA patients, IL-6 levels in synovial fluid were significantly higher than those in plasma and correlated positively with MMP enzymes (Figure 2) and radiographic severity of OA; while plasma IL-6 concentrations in OA patients were comparable to those reported in healthy individuals [2]. The importance of IL-6 in OA is also supported by previous results. It was shown that IL-6 concentrations in synovial fluid were considerably higher in patients with cartilage defect or OA than in donors without joint pathology [14,15]. Follow-up study showed that increased serum concentrations of IL-6 were associated with articular changes observed in radiographs [16]. In our study, advanced radiographic severity of OA was associated with higher IL-6 concentrations in synovial fluid but not in plasma. Clinical observations together with the present findings strongly support the important role of IL-6 in the pathogenesis of and as a potential drug target in OA.

Stilbenoids are naturally occurring compounds found in grapes, almond, rhubarb and berries which makes them part of our normal diet. Stilbenoids are also secondary products of heartwood formation in trees where they act as phytoalexins. Two silbenoids monomethyl pinosylvin and pinosylvin isolated from the knots of Scots pine (*Pinus sylvestris*) were identified to have anti-inflammatory potential in our previous studies [9]. Therefore we aimed to study their effects on chondrocytes, cell type significantly involved in the pathogenesis of OA. 

To our knowledge, the effects of monomethyl pinosylvin and pinosylvin have not previously been studied in OA. Whereas another stilbenoid, resveratrol, has been studied in arthritis models. In a surgically induced OA model in mice, resveratrol decreased destruction of articular cartilage, production of the catabolic factor MMP-13 and expression of the inflammatory enzyme iNOS [17]. Furthermore resveratrol increased thickness of the calcified cartilage and improved Mankin scores [17]. In another study, Mankin score improval and inhibition of cartilage destruction was seen in a surgical model of OA in rabbits after intra-articular resveratrol treatment [18]. Interestingly, resveratrol was found effective also in the prevention of collagen-induced arthritis model in mice [19]. Incidence and severity of arthritis as well as the amount of infiltrated cells in the joint were decreased after 8 weeks treatment with resveratrol and, cartilage and bone erosions and synovial hyperplasia were prevented [19]. 

In the present study, the pine stilbenoids monomethyl pinosylvin and pinosylvin, as well as resveratrol, were found to suppress IL-6 expression in primary OA chondrocytes stimulated with IL-1β or IL-17 (Figure 3). There is a very limited amount of data available of other stilbenoids than resveratrol in chondrocytes but resveratrol has been shown to reduce MMPs and IL-6 in human chondrocyte cultures supporting our finding [11,20]. Monomethyl pinosylvin and pinosylvin suppressed IL-6 expression possibly via a mechanism involving the inhibition of NF-κB activity (Figure 4). Both pine stilbenoids inhibited NF-κB mediated transcription in human chondrocyte cell line and NF-κB is a known transcription factor regulating IL-6 production [1].

The physical function of joints and biochemical properties of cartilage are critically reliant on the integrity of the extra cellular matrix (ECM). In normal conditions, articular chondrocytes preserve a dynamic balance between degradation and synthesis of ECM components. ECM is composed of a collagenous network, mostly type II collagen, alongside with glycosaminoglycans like hyaluronan, and a variety of proteoglycans including aggrecan. In OA, on the other hand, the equilibrium has been disrupted and catabolic processes are accelerated while anabolic processes are suppressed. [21]

Aggrecan mRNA expression was upregulated by monomethyl pinosylvin, pinosylvin and resveratrol in unstimulated and IL-1β or IL-17 stimulated primary human chondrocytes (Figure 5). The effect is beneficial to the cartilage homeostasis. Aggrecan as a component of proteoglycans is essential to maintain the normal function of articular cartilage because it draws water into cartilage matrix and forms a hydrated gel structure that provides the cartilage with load-bearing properties [21]. The increased aggrecan expression might also be a positive consequence of IL-6 inhibition because IL-6 has been shown to suppress proteoglycan production in murine bone marrow-derived mesenchymal stem cells [22]. There is also a previous study where resveratrol increased aggrecan expression similar to in our study but contrary to our study it also increased the collagen II expression [16]. One possible explanation for the differing result might be that they used non-arthritic articular cartilage to isolate primary chondrocytes while we had OA cartilage. 

It has been proposed that OA is complicated with IL-6 induced oxidative stress. IL-6 together with IL-1 dysregulates the antioxidant defense mechanisms in chondrocytes and increases the production of reactive oxygen species (ROS) [23,24]. ROS mediate intracellular events and regulate gene expression including MMPs supporting the degradation of cartilage matrix [25,26]. Free radicals can also attack directly proteoglycan and collagen molecules in ECM [26]. Thereby inhibition of mechanisms (such as IL-6) able to trigger ROS production and suppress antioxidant defense could be a reasonable target to prevent or treat OA.

Our results suggest that monomethyl pinosylvin and pinosylvin may have disease-modifying properties in OA chondrocytes through down-regulation of IL-6 and up-regulation of aggrecan. Interestingly, it has been recently reported that monomethyl pinosylvin inhibits TRPV1 activator capsaicin induced pain behavior [27] and that pinosylvin suppresses TRPA1-mediated ion currents in vitro and TRPA1-mediated acute paw inflammation in mice [28]. Therefore, it is tempting to speculate that in addition of being possible disease-modifying OA drug candidate stilbenoids might have also pain relieving properties in OA. 

In conclusion, the present findings indicate that IL-6 is produced within OA joints, and it is associated with increased levels of cartilage degrading MMP enzymes and with the severity of radiographically detected joint changes in patients with OA. For the first time monomethyl pinosylvin and pinosylvin were shown to inhibit IL-6 production and increase aggrecan expression in primary human OA chondrocytes. The results suggest an important role for IL-6 in the pathogenesis of OA and the potential of pine stilbenoids as disease-modifying compounds in OA chondrocytes.

## 4. Materials and Methods

### 4.1. Chemicals

Pinosylvin and monomethyl pinosylvin were obtained from Arbonova (Turku, Finland) and resveratrol from Tocris Bioscience (Ellisville, MS, USA). All other reagents were from Sigma Chemical Co (St. Louis, MO, USA) unless otherwise stated.

### 4.2. Patients and Clinical Samples

The patients fulfilled the American College of Rheumatology classification for OA [29]. Blood and synovial fluid samples were obtained from 100 OA patients [BMI 29.7 (8.3) kg/m^2^, age 72 (14) years, median (IQR); 62/38 females/males] undergoing total knee replacement surgery. Plasma and synovial fluid samples were stored at −80 °C until analyzed. The study was approved by the Ethics Committee of Tampere University Hospital, Tampere, Finland (ethics code R06223), and was conducted in accordance with the Declaration of Helsinki. All patients provided their written informed consent.

### 4.3. Primary Chondrocyte Experiments

Primary chondrocyte experiments were performed as previously described by Koskinen et al. [30]. Briefly, leftover pieces of OA cartilage from knee joint replacement surgery were used under full patient consent and approval by the Ethics Committee of Tampere University Hospital, Tampere, Finland (ethics code R09116). Full-thickness pieces of articular cartilage from femoral condyles and tibial plateaus showing macroscopic features of early OA were removed aseptically from subchondral bone with a scalpel, and cut into small pieces. Cartilage pieces were washed with PBS and chondrocytes were isolated by enzymatic digestion for 16 h at 37 °C in a shaker by using a collagenase enzyme blend (1 mg/mL Liberase Research Grade medium; Roche, Mannheim, Germany). Isolated chondrocytes were washed and plated on 24-well plates (1.5 × 105 cells/mL) in culture medium (DMEM with glutamax-I Gibco supplemented with penicillin (100 U/mL), streptomycin (100 μg/mL), and amphotericin B (250 ng/mL) (all from Thermo Fisher Scientific, Carlsbad, CA, USA) containing 10% fetal bovine serum (Lonza, Verviers Sprl, Verviers, Belgium). Samples for each primary chondrocyte experiment were obtained from three consequent donors and the cell culture experiments were performed in duplicate; *n* = 3 was used in the calculations. All measured values were included for the final results. Chondrocytes were stimulated with IL-1β (100 pg/mL) or IL-17 (50 ng/mL) both from R&D Systems Europe Ltd. (Abingdon, UK) with and without the tested compounds for 24 h. The culture media were stored at −20 °C until analyzed. Cytotoxicity of the investigated compounds was ruled out by measuring cell viability using Cell Proliferation Kit II (Roche) according to the manufacturer’s instructions.

### 4.4. Preparation of the Stable T/C28a2pGL4.32NFκB Cell Line

In order to investigate the effects of the stilbenoids on the NF-κB mediated transcription, the T/C28a2 human chondrocyte cell line kind gift from Professor Mary B. Goldring were stably transfected with luciferase reporter construct, pGL4.32[luc2P/NF-κB-RE/Hygro] [31]. The plasmid was purchased from Promega Corporation (Madison, WI, USA) and contains five copies of an NF-κB response element that drives transcription of the luciferase reporter gene. T/C28a2 human chondrocyte cell line was cultured at 37 °C in 5% CO_2_ atmosphere and grown in DMEM/Ham’s F12 (1:1) containing 5% heat-inactivated foetal bovine serum, penicillin (100 units/mL), streptomycin (100 μg/mL) and amphotericin B (250 ng/mL). Cells were seeded on 24 well plates and cell monolayers were grown for 72 h to confluence before the experiments were started and the compounds of interest were added in fresh culture medium. Firefly luciferase activity was measured using the luciferase assay reagent (Promega Corp., Madison, WI, USA), and the results were normalized to the total cellular protein.

### 4.5. Measurement of IL-6 and MMPs by Immunoassay

Concentration of IL-6 in plasma, synovial fluid, and culture media was measured by enzyme-linked immunosorbent assay (ELISA) with commercial reagents from Sanquin (Amsterdam, The Netherlands). The detection limit for IL-6 was 0.3 pg/mL. MMP-1 concentrations in the synovial fluid were determined by Multiplex bead array (Fluorokine^®^ Human MMP Multi Analyte Profiling Base Kit, R&D Systems, Inc., Minneapolis, MN, USA) and MMP-3 concentrations were assessed by ELISA (R&D Systems, Inc.). Detection limits were 10.7 pg/mL for MMP-1 and 15.6 pg/mL for MMP-3. 

### 4.6. Measurement of IL-6, Collagen II and Aggrecan mRNA Levels

At the indicated time points, culture medium was removed from primary human OA chondrocytes and total RNA was extracted with GenElute™ Mammalian Total RNA Miniprep Kit (Sigma-Aldrich, St Louis, MO, USA) according to the manufacturer’s instructions. Total RNA was reverse-transcribed to cDNA using TaqMan Reverse Transcription reagents and random hexamers (Applied Biosystems, Foster City, CA, USA). cDNA obtained from the RT-reaction was diluted 1:20 with RNAse-free water and subjected to quantitative PCR using TaqMan Universal PCR Master Mix and ABI PRISM 7000 Sequence detection system (Applied Biosystems). Primers and probes (Table 1) for IL-6, aggrecan, collagen II and glyceraldehyde-3-phosphate dehydrogenase (GAPDH, used as a control gene) were designed using Primer Express^®^ Software (Applied Biosystems) and supplied by Metabion (Martinsried, Germany). 

The primer and probe sequences and concentrations were optimized according to manufacturer’s guidelines in TaqMan Universal PCR Master Mix Protocol part number 4,304,449 revision C. PCR reaction parameters were as follows: incubation at 50 °C for 2 min, incubation at 95 °C for 10 min, and thereafter 40 cycles of denaturation at 95 °C for 15 s and annealing and extension at 60 °C for 1 min. A standard curve method was used to determine the relative mRNA levels.

### 4.7. Statistical Analysis

SPSS program version 17.0 for Windows software (SPSS Inc, Chicago, IL, USA) was used for analyzing clinical data. Normality of the data was tested by Kolmogorov-Smirnov test. Based on that, nonparametric tests were used in the analysis. Differences between groups were tested by Wilcoxon Signed Rank Test. Pearson’s r was used to analyse correlation after natural logarithm (LN) transformation by which normal distribution was achieved. P-values less than 0.05 were considered significant. 

Chondrocyte results are expressed as the mean ± standard error of mean (SEM). Statistical significance of the results was calculated by one-way ANOVA with Bonferroni’s post-test by using GraphPad InStat 3 for Windows XP (Graph-Pad Software, San Diego, CA, USA). Differences were considered significant at * *p* < 0.05, ** *p* < 0.01 and *** *p* < 0.001. 

## Figures and Tables

**Table 1:** Primer and probe sequences.

Gene	Oligonucleotide	Sequence 5′→3′
Human	Forward primer	TACCCCCAGGAGAAGATTCCA
Interleukin-6	Reverse primer	CCGTCGAGGATGTACCGAATT
	Probe	CGCCCCACACAGACAGCCACTC
Human	Forward primer	GGCAATAGCAGGTTCACGTACA
Collagen II	Reverse primer	CGATAACAGTCTTGCCCCACTT
	Probe	CTGAAGGATGGCTGCACGAAACATACC
Human	Forward primer	GCCTGCGCTCCAATGACT
Aggrecanase	Reverse primer	TAATGGAACACGATGCCTTTCA
	Probe	CCATGCATCACCTCGCAGCGGTA
Human	Forward primer	AAGGTCGGAGTCAACGGATTT
GAPDH *	Reverse primer	GCAACAATATCCACTTTACCAGAGTTAA
	Probe	CGCCTGGTCACCAGGGCTGC

* GAPDH: Glyceraldehyde 3-phosphate dehydrogenase.
